# Hepatic Portal Venous Gas: Comparison of Two Cases

**DOI:** 10.1155/2013/637951

**Published:** 2013-10-07

**Authors:** Iain Rankin, Hemant Sheth

**Affiliations:** Department of Upper GI and Laparoscopic Surgery, Ealing Hospital NHS Trust, London, Uxbridge Road, Southall, UB1 3HW, UK

## Abstract

*Context.* Hepatic portal venous gas (HPVG) is a rare and sinister finding. Its mortality is associated with the underlying causative condition. When secondary to bowel ischaemia, mortality rates exceed 50%. *Case Report.* Two cases of HPVG are described. One case describes HPVG in association with gastric ischaemia, with complete resolution following conservative management. The second case describes HPVG in association with widespread intra-abdominal ischaemia, with resultant mortality. *Conclusion.* A “watch and wait” management of HPVG associated with gastric ischaemia is suggested in certain patients, with a low threshold for surgical intervention. HPVG associated with bowel ischaemia is an absolute indication for surgical intervention, where intervention may change the clinical course.

## 1. Introduction

Hepatic portal venous gas is a rare and sinister finding. It is associated with numerous underlying abdominal pathologies, ranging from benign conditions that require no invasive treatment to potentially lethal diseases that necessitate prompt surgical intervention. When secondary to bowel ischaemia, mortality rates exceed 50% [1]. We describe two cases of HPVG associated with intra-abdominal ischaemia.

## 2. Case 1

An 81-year-old man of Indian descent presented to our emergency department with severe abdominal pain. The pain was localised to the right upper quadrant and epigastrium, radiated to his back, came on suddenly, and had worsened over the previous 12 hours. His previous medical history included angina, hypertension, type II diabetes mellitus, glaucoma (registered blind), and recurrent postprandial abdominal pain for which he received telmisartan, bendroflumethiazide and latanoprost eye drops. The patient denied any alcohol intake or smoking. He was febrile (38.3°C), tachycardic, and tachypnoeic with other observations within normal limits. On examination, he had generalised abdominal pain with severe tenderness in the epigastric and right upper quadrant regions. There were no signs of peritonism and bowel sounds were normal.

Full serological testing was performed which revealed a deranged liver profile (bilirubin 31 umol/L, alanine transaminase 519 IU/L and gamma glutamyl transferase 565 IU/L), deranged renal profile (urea 9.5 mmol/L, creatinine 171 umol/L), a raised amylase of 2197 IU/L, and a metabolic acidosis; inflammatory markers were mildly raised and other parameters were unremarkable.

**Figure 1:**
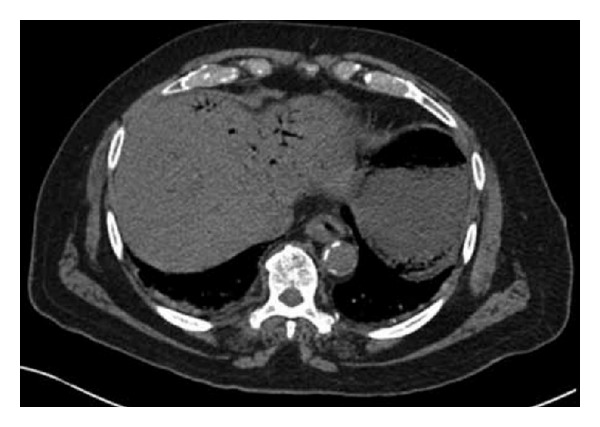
CT showing hepatic portal venous gas (Case 1).

**Figure 2:**
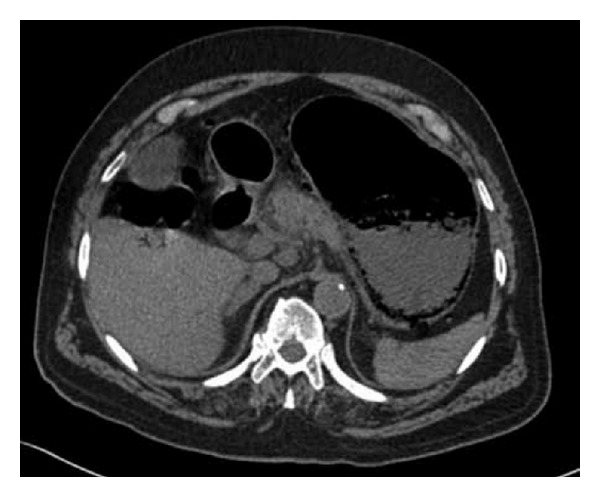
CT showing gas within the posterior gastric wall, indicative of gastric ischaemia (Case 1).

**Figure 3:**
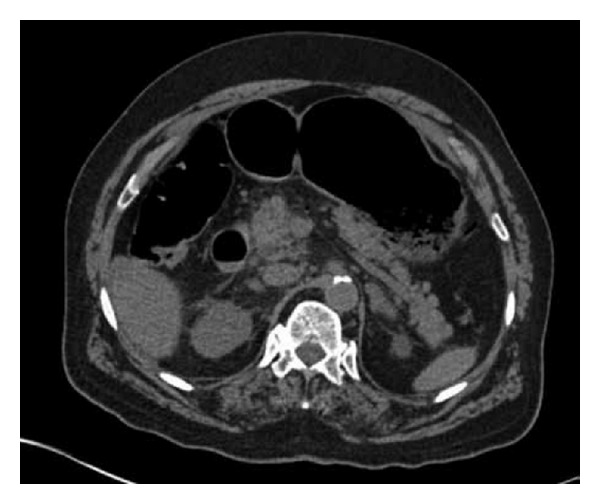
CT showing a calcified plaque at the origin of the celiac axis (Case 1).

Erect chest X-ray and abdominal X-ray showed no pathology. With signs of an acute abdomen and diagnostic doubt, a computerised tomography (CT) scan was carried out. Due to the patient's elevated creatinine, a noncontrast CT scan was performed. CT revealed extensive hepatic portal venous gas. Branching air filled structures within the liver extending to the periphery, representing portal venous radicles, were noted (Figure 1). Furthermore, gas was seen within several upper abdominal mesenteric veins and within the posterior gastric wall, indicative of gastric ischaemia (Figure 2). A calcified plaque was noted at the origin of the celiac axis, believed to be the causative factor for the patient's recurrent postprandial abdominal pain and current clinical picture (Figure 3).

**Figure 4:**
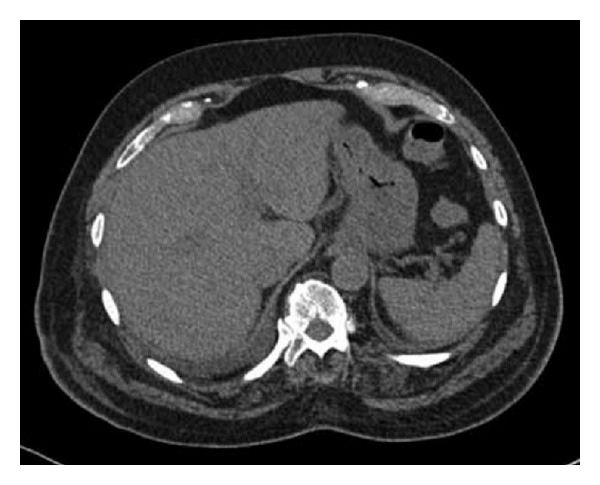
CT showing resolution of the hepatic portal venous gas (Case 1).

Following discussion with the regional upper gastrointestinal tertiary centre, due to extensive comorbidities, the patient was deemed unsuitable for surgery. The patient was managed conservatively by commencement of antibiotic therapy (piperacillin with tazobactam) and a proton pump inhibitor (IV pantoprazole). Three days following the initiation of treatment, clinical improvement was noted and a repeat CT of abdomen was carried out. CT revealed significant interval improvement—no intraabdominal gas was seen (Figure 4).

The patient completed a two-week course of piperacillin with tazobactam. He remained asymptomatic, biochemical markers improved and he was discharged with outpatient follow-up. Endoscopy was carried out five weeks later demonstrating no abnormality. The patient was followed up in clinic for over a year, remaining asymptomatic throughout.

## 3. Case 2

A 74-year-old Caucasian woman presented to our emergency department with abdominal pain, bleeding per rectum, diarrhea, and vomiting. She reported a three-day history of generalised abdominal pain, vomiting and diarrhoea. In the morning of presentation, she had noticed a bright red PR bleed mixed with stool. She reported no fevers or recent travel. Her previous medical history included pancreatic insufficiency, diverticular disease, recurrent ischiorectal abscess, previous pseudomonas colitis, previous peptic ulcer, previous myocardial infarction, hypertension, hypercholesterolaemia, peripheral vascular disease, right femoral pseudoaneurysm, aortobifemoral graft, and chronic obstructive pulmonary disease. Her medications included pancrelipase, clopidogrel, bisoprolol, isosorbide mononitrate, omeprazole, ramipril, and simvastatin. The patient admitted to smoking 15 cigarettes a day and denied any alcohol consumption. She was afebrile, tachycardic, and tachypnoeic with other observations within normal limits. On examination, she had generalised abdominal tenderness. A pulsatile mass was felt in the right groin. There was no signs of peritonism and bowel sounds were normal.

Full serological testing was performed which revealed a deranged renal profile (urea 33.4 mmol/L, creatinine 225 umol/L, potassium 3.2 mmol/L), deranged liver profile (alanine aminotransferase 117 IU/L), raised inflammatory markers (white cells 33.4 × 10^9^/L, C-reactive protein 131 mg/L), and metabolic acidosis; other parameters were unremarkable. Stool cultures returned with no growth and were negative for clostridium difficile toxin, ova, cysts, and parasites.

Erect chest X-ray and abdominal X-ray revealed no pathology. CT revealed an old pseudoaneurysm of the right groin with aortobifemoral graft, no cause for the patient's abdominal pain and diarrhoea identified. 

The patient was given a short course of the antimicrobials cefuroxime and metronidazole and received regular intravenous fluid with potassium replacement. She began to improve both clinically and biochemically. 

Whilst undergoing treatment, the patient experienced an episode of chest pain. ECG and cardiac enzymes confirmed a non-ST-elevation MI with new onset of atrial fibrillation. The patient was treated according to a standardized treatment protocol and scheduled for angiogram following the cessation of sepsis. 

Further to this event, the patient complained of right upper quadrant pain. Serological testing revealed a sharp rise in inflammatory markers (white cells of 34.2 × 10^9^/L, 11.8 × 10^9^/L the previous day) with markedly deranged liver profile (alkaline phosphatase 604 IU/L, alanine aminotransferase 113 IU/L). Abdominal USS was carried out. The liver was visualised with normal size, echogenicity, and contour. Doppler imaging showed normal portal vein flow. Adenomyomatosis of the gallbladder was noted and the abdominal USS was otherwise unremarkable. Chest X-ray, blood culture, urine culture, and stool culture at this time showed no pathology. Whilst vomiting had now been resolved, the patient had experienced continuing intermittent diarrhoea during admission. The patient was started on the antimicrobial piperacillin with tazobactam for a sepsis of unknown origin. A repetition of chest X-ray several days later confirmed a pneumonia of the right lower lobe. Repeat blood cultures returned positive for Citrobacter koseri sensitive to current antimicrobials. Her abdominal pain had now settled. Inflammatory markers, however, showed minimal improvement to the current antimicrobial regime. 

CT of the chest, abdomen, and pelvis was carried out. CT confirmed an extensive right lower lobe pneumonia. CT also showed new intrahepatic bile duct dilatation. No obstructive lesion or calculi were visualised. The patient was scheduled for MRCP. The antimicrobial regime was continued and serological testing showed a gradual improvement in inflammatory markers.

Subsequent to this, the patient began to experience severe abdominal pain. The pain had come on suddenly, was maximal within the epigastric region, and had worsened since onset. It was associated with vomiting and a fresh PR bleed of 200 mL was reported. On examination, generalised abdominal tenderness with localised tenderness in the epigastric region was found. The abdomen was rigid with guarding and rebound tenderness. No bowel sounds were heard. Observations revealed a tachycardia. Blood pressure, oxygen saturation, respiratory rate and temperature were within normal limits. Oliguria was noted. An arterial blood gas was carried out, revealing a 1.4 g/dL drop of haemoglobin since previous serological testing that morning and a compensated metabolic acidosis, lactate 7.1 mmol/L. A differential diagnosis of ischaemic bowel or bowel perforation was made. Blood transfusion was commenced, the antimicrobials teicoplanin and gentamicin were added, and intravenous vitamin K and intravenous pantoprazole were given.

**Figure 5:**
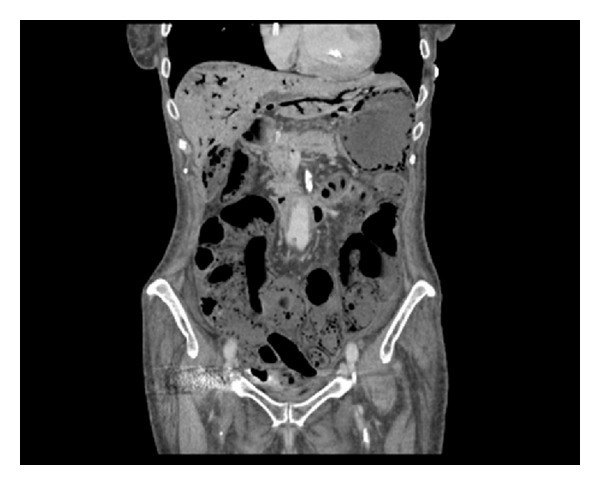
CT showing extensive hepatic portal venous gas, gas within the stomach wall and its draining veins, the gallbladder, common bile duct, and within the majority of the walls of the small bowel (Case 2).

**Figure 6:**
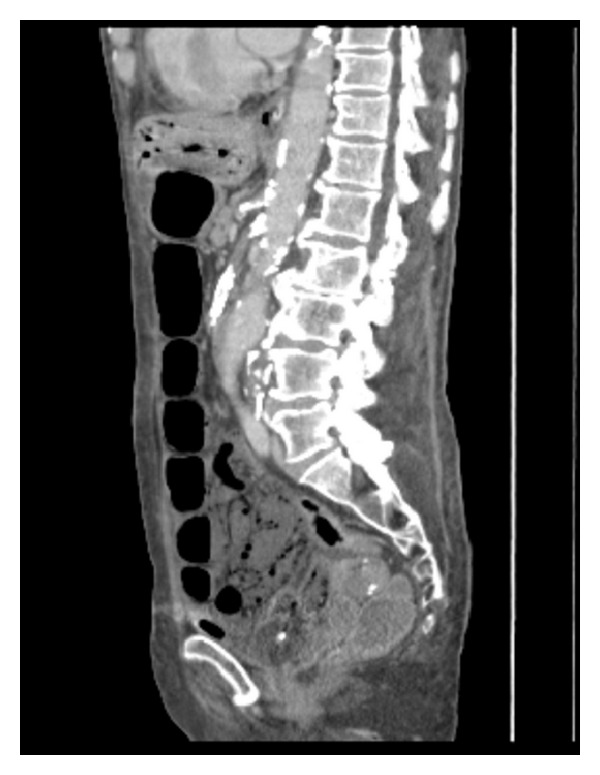
CT showing nonenhancement of the superior mesenteric artery following contrast. The celiac axis is heavily calcified with minimal contrast enhancement (Case 2).

Subsequent urgent CT abdomen with contrast was carried out, revealing evidence of widespread bowel ischemia. Gas was seen within the portal vein with extensive gas noted within the liver, within the stomach wall and its draining veins, the gallbladder, common bile duct, and within the majority of the walls of the small bowel (Figure 5). The superior mesenteric artery did not enhance following contrast. The celiac axis was heavily calcified throughout with minimal contrast enhancement (Figure 6).

Due to the degree of widespread ischaemia, no surgical intervention was carried out and conservative management continued. The patient died 36 hours later. 

## 4. Discussion

Hepatic portal venous gas (HPVG) is a rare and sinister radiological sign. It was first described by Wolfe and Evans in 1955 in infants with necrotizing enterocolitis [2] Subsequent review of adult cases in 1978 found an associated mortality of 75% [3]. With the advent of computerised tomography, more cases of HPVG have been identified. Nevertheless, it is still a rare finding, with only 182 cases documented in the literature by 2001 [4]. Advanced imaging has detected smaller volumes of HPVG not detectable on X-ray, allowing for earlier intervention and incidental findings of HPVG in benign conditions. As such, a review in 2001 found a reduced total mortality of 39% [4]. When HPVG is accompanied by small bowel ischaemia, mortality remains high, with rates in excess of 50% [1].

The exact pathophysiology of HPVG is unclear and several theories have been hypothesised. The escape of microbe-derived gas production from the bowel lumen or in an abscess which then circulates into the liver and the presence of gas forming organisms within the portal system are proposed factors [3]. No clear data exists to describe how gas production secondary to microbial metabolism results in HPVG [5]. Another leading hypothesis is of absorbed intraluminal air. An impaired epithelial barrier, by means of ischaemic bowel, or an increased intraluminal pressure could result in entry of luminal air into the capillary veins [1].

The diagnosis of HPVG can be made by plain abdominal X-ray, ultrasound with Doppler imaging or CT. Abdominal X-ray will only detect significant volumes of HPVG—if present, mortality is in excess of 50% [1]. Ultrasound with Doppler is cost effective, noninvasive, and highly sensitive and can be useful as a first line of investigation [6, 7]. Ultrasound is operator dependent and so provides variable outcomes, limiting its use. CT provides a high sensitivity for HPVG and can reveal the underlying pathology [8, 9]. The prognosis of HPVG is related to the pathology itself and is not influenced by the presence of HPVG [8].

HPVG is associated with numerous underlying abdominal pathologies, ranging from benign conditions that require no invasive treatment to potentially lethal diseases that necessitate prompt surgical intervention [4, 10–12]. Bowel ischaemia and/or infarction is still the most common underlying cause and the most important diagnosis to exclude due to its associated high mortality, with rates reported as high as 75–90% [3, 13]. Transmural necrosis is present in 91% of cases [13]. In our second case, clinical findings suggesting bowel ischaemia were accompanied by CT findings of HPVG and pneumatosis intestinalis. In cases with these findings the prognosis is poor [14–18]. As such, HPVG associated with bowel ischaemia is an absolute indication for surgery, with intestinal resection of necrotic bowel. In our second case, the bowel ischaemia was so extensive that surgical intervention would not alter the clinical course.

The first case we have presented is a patient with HPVG due to gastric ischaemia. It has previously been recommended that patients with acute gastric ischaemia undergo partial or total gastrectomy [19]. In our case, conservative management resulted in complete resolution of symptoms and pathology with continuing resolution at 1-year follow-up. Our patient described a history of recurrent postprandial abdominal pain, a likely manifestation of chronic gastric ischaemia secondary to stenosis of his celiac axis. The chronic nature of his gastric ischaemia may have allowed development of improved collateral circulation. The stomach has a rich vascular supply consisting of five major vessels, minor vessels, and collateral supply. It has been previously demonstrated in animal models that gastric necrosis would require the ligation of the left and right gastric arteries, left and right gastroepiploic arteries, and over 80% of collaterals [20]. Cadaveric studies have shown that only one patent major vessel is required to provide complete gastric wall vascular filling [21]. Previous authors have similarly reported the complete reversal of gastric ischaemia in a patient following conservative management only [22]. This suggests a “watch and wait” approach may be taken in patients with gastric ischaemia where no evidence of peritonism or haemodynamic compromise is present. A low threshold for surgical intervention should be adopted if lack of improvement or clinical deterioration occurs.

In conclusion, we have reported two cases of HPVG. One case was associated with only gastric ischaemia and showed complete reversibility with conservative management. The second case was associated with widespread intra-abdominal ischaemia and resultant mortality occurred. The first case suggests the possibility of a “watch and wait” management of HPVG associated with gastric ischaemia in certain patients; the second case highlights the high mortality rate of HPVG associated with bowel ischaemia and the necessity for surgical intervention, where intervention may change the clinical course.
